# Long-Term Efficacy of Intranasal Esketamine in Treatment-Resistant Major Depression: A Systematic Review

**DOI:** 10.3390/ijms22179338

**Published:** 2021-08-28

**Authors:** Enrico Capuzzi, Alice Caldiroli, Martina Capellazzi, Ilaria Tagliabue, Matteo Marcatili, Fabrizia Colmegna, Massimo Clerici, Massimiliano Buoli, Antonios Dakanalis

**Affiliations:** 1 Psychiatric Department, Azienda Socio Sanitaria Territoriale Monza, via G.B. Pergolesi 33, 20900 Monza, Italy; e.capuzzi1@campus.unimib.it (E.C.); m.marcatili@asst-monza.it (M.M.); f.colmegna@asst-monza.it (F.C.); massimo.clerici@unimib.it (M.C.); 2 Department of Medicine and Surgery, University of Milano Bicocca, via Cadore 38, 20900 Monza, Italy; m.capellazzi@campus.unimib.it (M.C.); i.tagliabue5@campus.unimib.it (I.T.); antonios.dakanalis@unimib.it (A.D.); 3 Department of Pathophysiology and Transplantation, University of Milan, via Festa del Perdono 7, 20122 Milan, Italy; massimiliano.buoli@unimi.it; 4 Department of Neurosciences and Mental Health, Fondazione IRCCS Ca’ Granda Ospedale Maggiore Policlinico, via F. Sforza 35, 20122 Milan, Italy

**Keywords:** treatment-resistant depression, esketamine, clinical trials, continuation, systematic review

## Abstract

Esketamine (ESK) has been approved as a rapid-acting intranasal treatment for treatment-resistant depression (TRD). Although existing studies have investigated the efficacy of ESK in the 4-week induction phase, our knowledge about long-term ESK efficacy remains poor. The aim of this systematic review was to summarize the available data on long-term ESK efficacy for TRD. A systematic search was performed including articles in English, up to 31 March 2021. The search found 7 relevant studies, involving 1024 adult TRD patients. Continuing treatment with ESK after the 4-week induction phase may be associated with stable efficacy in relapse prevention among TRD patients. Conversely, the long-term antidepressant effectiveness upon discontinuation of ESK might be limited, although data from three studies had a moderate to high risk of bias. Overall, the results on the effectiveness of this compound in the long term are mixed. According to our findings, ESK treatment should be continued following the induction phase to reach a stable efficacy in relapse prevention, while the long-term antidepressant and anti-suicidal effects of ESK after discontinuation are inconsistent. Currently, the level of proof of ESK efficacy in long-term TRD treatment remains low and more RCTs with larger sample sizes and active comparators are needed.

## 1. Introduction

Major depressive disorder (MDD) is one of the most common and disabling mental disorders. Although several (monoaminergic) antidepressants (ADs) exist, the therapeutic response is often partial and around 30% of MDD patients develop treatment-resistant depression (TRD) [1], i.e., they do not respond to at least two adequate AD treatments [2], with the related sequelae in terms of worse prognosis [3,4]. Thus, new and fast-acting molecular targets outside the monoaminergic system [5,6] are needed to engender clinically meaningful advances in MDD therapeutics. Post-mortem analyses, in vivo gene expression studies and brain imaging data suggest abnormalities in glutamatergic signaling in MDD pathophysiology [7,8].

Ketamine, an old anesthetic acting as a glutamate N-methyl-D-aspartate receptor (NMDAR) antagonist, has shown significant AD effects in both human and animal models of MDD [9,10]. As a result of its specific pharmacodynamic properties [11], ketamine addresses some of the unmet needs of MDD treatment, with a rapid onset of action [12], high response rates in TRD [13] and reduction of suicidal ideation [14]. Nevertheless, adverse events have been reported in different studies [15,16] and the intravenous formulation of ketamine may hamper its administration in several clinical settings [17]. In this framework, esketamine (ESK), the S-enantiomer of ketamine, had about a fourfold greater affinity for the NMDAR than ketamine, thus allowing the use of much lower doses and reducing the risk of dose-dependent dissociative symptoms associated with ketamine [18] administration. On 5 March 2019, the nasal-spray formulation of ESK was approved by the Food and Drug Administration (FDA) after showing its efficacy in TRD patients [19].

Recent meta-analyses confirm the rapidity and efficacy of ESK in patients with TRD [20,21] or at imminent risk for suicide [22] although a significant rate of patients discontinue the treatment for poor tolerability [21]. Although many studies focus on the short-term efficacy of ESK [23,24,25], namely the 4-week induction phase, our knowledge about the long-term effect of this compound is poor, both in patients who discontinued the treatment after the induction phase and those who continued the AD.

No systematic review of long-term efficacy of intranasal ESK has been published so far. This paper critically summarizes the available data on ESK long-term effects in TRD patients after the 4-week induction phase.

## 2. Results and Discussion

The search provided a total of 438 citations. Among these, 236 were identified as duplicates; 202 studies were screened and, after reviewing the abstracts, 177 were discarded. In particular, 52 papers dealt with a different topic, 2 were conducted on animals, and 5 were not written in English. Further, 7 studies were excluded because they were still ongoing, 68 were reviews or meta-analyses and 37 were comments, case reports or letters. Six papers were discarded as they consisted of study design, proof of concept or study protocols.

**Figure 1:**
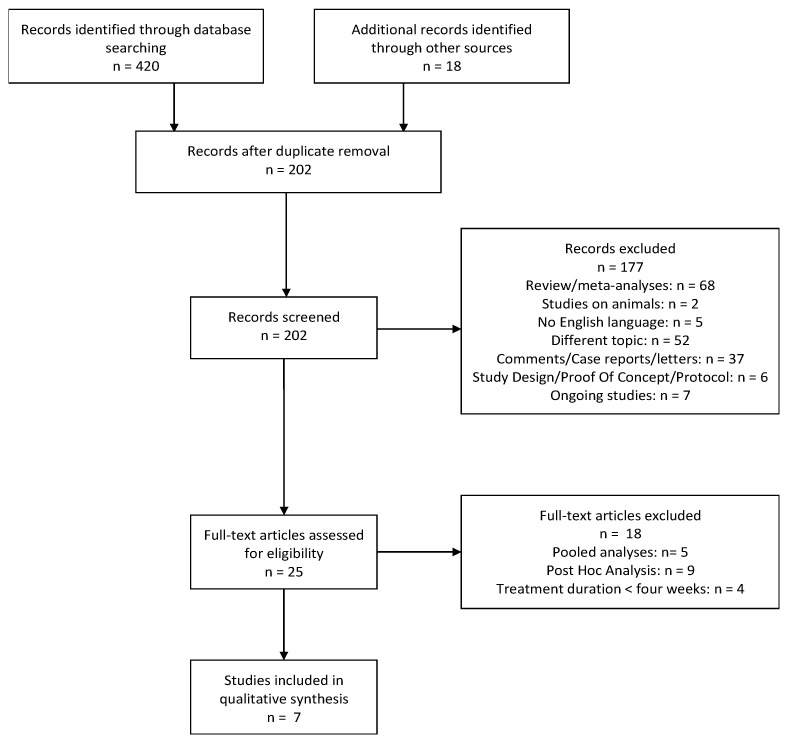
PRISMA flowchart for systematic reviews.

The full texts of the remaining 25 citations were examined in more detail, and we concluded that 18 studies did not meet the inclusion criteria because 5 were pooled analyses, 9 were post-hoc analyses and 4 reported a duration of ESK treatment shorter than 4 weeks. Thus, 7 studies met the inclusion criteria and were included in the present review (Figure 1).

### 2.1. Esketamine Treatment Continuation after the 4-Week Induction Phase

The multicenter phase-II placebo-controlled RCT (randomized clinical trial) NCT01998958 [26] originally screened 126 patients with TRD (defined as inadequate response to two or more ADs, with at least one not appropriate response in the current depressive episode). The study design consisted of 4 periods: (1) screening, (2) a double-blind treatment (days 1–15 with 3:1:1:1 randomization to placebo, intranasal ESK 28 mg, 56 mg or 84 mg twice weekly), (3) an optional open-label treatment (days 15–74 during which intranasal dosing frequency of ESK was tapered with administration twice weekly for the first 2 weeks, weekly for the next 3 weeks, and then every 2 weeks) and (4) an 8-week post-treatment follow-up phase. All included patients continued to take their previous AD during the trial and started or continued ESK during the optional open-label phase. Exclusion criteria consisted of suicidal or homicidal ideation or intent, suicidal behavior, several psychiatric comorbidities (psychotic disorders, MDD with psychotic features, substance use disorders (SUD) and bipolar disorder (BD)). A total of 57 patients started the open-label phase, although only 40 of them completed the 8-week final follow-up phase. The patients ending the final follow-up phase benefited from remarkable remission/response rates, therefore the authors concluded that patients treated with ESK ameliorated their depressive symptoms in the long term, even after discontinuation of the compound.

A multicenter phase-III double-blind RCT called SUSTAIN-1 (NCT02493868) [27] evaluated the efficacy of ESK plus AD in comparison to AD plus intranasal placebo among TRD outpatients (non-responders to at least 1 but no more than 5 ADs in the current moderate/severe depressive episode). The study design consisted of 5 phases: screening, 4-week open-label induction (ESK 56 mg or 84 mg twice weekly plus an AD), 12-week optimization (open-label or double-blind during which responders were treated with fixed doses progressively reduced to once weekly for 4 weeks, then to weekly or every 2 weeks), maintenance (double-blind, randomized withdrawal with variable duration during which patients with stable remission or response continued ESK treatment) and 2-week post-treatment follow-up. Exclusion criteria consisted of current or recent homicidal/suicidal ideation or intent as well as suicidal behavior in the previous year, moderate or severe SUD in the last 6 months, history of psychotic disorders, and diagnosis of MDD with psychotic features. Among 297 eligible patients, 176 and 121 individuals achieved, respectively, stable remission and response in the maintenance phase. Particularly, the relapse rate (MADRS total score ≥22 for two consecutive assessments) was lower among patients in treatment with ESK (both in those with stable remission or response) than placebo groups. Moreover, the patients receiving ESK benefited from a longer time to relapse compared to those receiving placebo.

Finally, another phase-III, open-label, multicenter study named SUSTAIN-2 (NCT02497287) [28] was originally conducted on 802 TRD outpatients (defined as non-responders to ≥2 AD during the current moderate/severe depressive episode). The sample was randomized to ESK plus AD or intranasal placebo plus AD. ESK plus AD was administered twice a week in a 4-week induction phase (at age-dependent flexible doses, 56 mg or 84 mg in individuals aged <65, and 28, 56 or 84 mg in patients ≥65 years) and, then, weekly or every other week for responders who entered a 48-week optimization/maintenance phase. Exclusion criteria were suicidal/homicidal ideation or intent, comorbidity with different psychiatric disorders (psychotic disorders, BD and MDD with psychotic features) and moderate/severe substance misuse. One hundred fifty patients completed the maintenance phase. The authors concluded that the improvement in MADRS, Patient Health Questionnaire-9 (PHQ-9) and Sheehan Disability Scale (SDS) scores may persist in individuals who continued ESK for up to 1 year.

Further details about these studies are summarized in Table 1.

### 2.2. Follow-Up Phase after the 4-Week Induction Phase

Five trials reported data on the efficacy of intranasal ESK after stopping treatment administered during the 4-week induction phase (Table 2).

Apart from the RCT NCT01998958 [26], only one RCT assessed the improvement in depressive symptoms after cessation of ESK among TRD patients [29]. The phase-II RCT (NCT02918318) [29], including 202 Japanese TRD patients with a single-episode or recurrent MDD without psychotic features and a MADRS total score ≥28, comprised a double-blind induction phase (days 1–28 with randomization to placebo or intranasal ESK 28 mg, 56 mg or 84 mg twice weekly in augmentation to oral AD treatment) and a post-treatment phase in which remitters (MADRS total score ≤12) and responders (participants who had ≥50% reduction from baseline in MADRS total score) were eligible. During the post-treatment phase, time to relapse was defined as the time (days) between induction phase and the first documentation of a relapse event during the post-treatment phase (MADRS total score ≥22 for two consecutive assessments). Exclusion criteria included homicidal ideation/intent, suicidal ideation in the 6 months before the screening phase, history of moderate or severe SUD. The authors reported that, among the 37 patients with clinical remission, time to relapse was higher in those taking ESK 56 mg than in those taking ESK 84 mg, ESK 28 mg or placebo. Alternatively, among the 68 responders, time to relapse was higher in patients taking placebo than in individuals receiving ESK 84 mg, ESK 28 mg or ESK 56 mg.

Three RCTs evaluated the possible efficacy of intranasal ESK among individuals with MDD and prominent suicidal risk.

A phase-II, placebo-controlled, multicenter RCT (NCT02133001) [30] compared the efficacy of ESK 84 mg with intranasal placebo in association with standard-of-care treatment. Sixty-eight individuals with a diagnosis of MDD without psychotic features (MADRS total score ≥22), responding affirmatively to Mini-International Neuropsychiatric Interview (MINI) about suicidal ideation/intent in the past 24 h (affirmative responses to “think about suicide?” and “intend to act on thoughts of killing yourself in the past 24 h?”) and requiring acute psychiatric hospitalization were eligible for the study. Exclusion criteria included diagnosis of BD, moderate to severe SUD, intellectual disability, antisocial and personality disorders and a current or past diagnosis of psychotic disorder. The study comprised an up to 48-h screening phase, followed by 4 weeks (days 1–25) of double-blind treatment and an 8-week follow-up (days 26–81). Participants taking their standard-of-care treatment were randomly assigned (1:1) to administer either intranasal placebo (*n* = 31) or intranasal 84 mg ESK (*n* = 35), twice weekly. During the follow-up phase, there was no improvement in MADRS total score between individuals belonging to ESK group and those treated with placebo. Similarly, authors reported no difference in the suicide thoughts assessed by the specific items of MADRS and the 21-item Beck Depression Inventory- BDI between the ESK and the placebo group.

The phase-III, multicenter RCT named ASPIRE I (NCT03039192) [31] was conducted to compare ESK 84 mg to placebo, in augmentation to standard-of-care treatment, among MDD subjects with suicidal ideation. Two hundred twenty-six patients with a diagnosis of MDD without psychotic features (MADRS total score ≥28), responding affirmatively to MINI questions about suicidal ideation/intention in the past 24 h and requiring admission to a psychiatric acute inpatient hospital, were included. Individuals with BD, obsessive-compulsive disorder, antisocial and personality disorders, moderate to severe SUD or current or prior diagnosis of psychosis were excluded. The study included a screening phase of 48 h, followed by a double-blind treatment phase (days 1–25) and a follow-up period (days 26–90). A total of 192 patients entered the follow-up phase whilst 164 completed the final follow-up visit at day 90. Authors reported that MADRS total scores were similar between ESK and placebo group and remained low throughout the follow-up period.

The subsequent identically designed phase-III, placebo-controlled, multicenter RCT named ASPIRE II (NCT03097133) [32], including 166 MDD patients with suicidal ideation, reported that both MADRS total scores and severity of suicidality (assessed through Clinical Global Impression of Severity of Suicidality Scale—CGI-SS Scale) remained low at the end of the follow-up period among subjects previously treated both with ESK 84 mg and placebo.

To the best of our knowledge, this is the first systematic review including studies exploring the efficacy of adjunctive intranasal ESK in the long term. Two main considerations can be made after the critical summary of the available data. First, according to the RCT by Daly and collaborators [26], including 57 patients and two large sample size RCTs for a total of 447 patients [27,28], the continuation of nasal ESK after the induction phase may favor clinical stabilization and relapse prevention in patients affected by TRD. Second, the long-term antidepressant effect of ESK upon discontinuation might be limited. Although a small-sample study by Daly and collaborators [26] reported that antidepressant activity of ESK might be maintained, contrasting results emerged from another larger RCT including 105 patients with TRD [29]. Similarly, initial clinical improvement might be lost after cessation of ESK in patients with MDD and current prominent suicidal risk. According to a phase II RCT including 44 patients [30] and two phase III RCTs totaling 330 individuals [31,32], no differences were found between ESK and placebo in terms of reduction of severity of depression and suicidal ideation in the post-treatment follow-up phase.

Although ESK seems to be effective in the long term only for continuation of treatment, some considerations are needed for an appropriate interpretation of the available data. First, patients in the included trials were largely unrepresentative of the real-world population with depression. All studies including participants with TRD excluded individuals with current suicide risk [26,27,28,29] whilst in both trials including individuals with TRD and prominent suicide risk [30,31,32], different psychiatric comorbidities including psychotic, bipolar, personality and obsessive-compulsive disorders and SUD were excluded. Notably, all studies enrolled medically stable patients. Furthermore, studies assessing individuals at elevated risk of suicide were conducted in the context of a comprehensive second-level standard of care, including initial hospitalization and optimization of oral antidepressant treatment. Second, some studies were based on a relatively small sample size with three trials [26,29,30] including less than 50 participants. Nevertheless, some trials enrolled individuals from a specific geographic area, i.e., United States and Japan [29,30]. Third, the quality of some studies may be influenced by the open-label study design [26,28,29] and the absence of a placebo or an active comparator group [28]. Moreover, according to the characteristic dissociative side effects of ESK, the blindness of studies could have been biased. Fifth, the rating scales used in the different studies to assess depressive symptoms and suicidal risk are heterogeneous. Nevertheless, the studies included in this review have different durations of both ESK treatment continuation and follow-up after the induction phase. Sixth, most of the included studies do not explicitly report relevant and useful data to evaluate the efficacy of ESK in the long term [26,28,30,31,32]. Some studies may be affected by moderate [28] to high risk of bias [26,30] as well as presenting some concerns in term of risk of bias [31,32]. A meta-analysis could not, therefore, be performed since effect estimates of included studies at risk of bias could be seriously misleading [33,34].

In addition to the high heterogeneity within the selected studies and inconsistency of some findings, two important aspects should be considered. Firstly, the clinical relevance of ESK use after the induction phase is not fully understood. TRD has a chronic course, poor clinical stabilization and high suicidal risk, and therefore to date it is difficult to know whether clinicians should continue to use ESK after the acute episode, for how long, at which dose and in which patients. As suggested by the results of the included trials, it might be argued that continuation of ESK after the induction phase may result in more clinical stabilization of patients with TRD. Moreover, another relevant question is how ESK should be discontinued. Even though ESK was safe and well tolerated even in the long term, potential abuse and effects on cognition should be taken into account, especially in the case of prolonged administration [15,35,36]. Another issue to be considered is the uncertain effect of ESK on suicidal ideation in the long term. ESK appeared to be a promising intervention in rapidly reducing suicidal ideation among patients with affective disorders [22] but the anti-suicidal mechanism of this compound should be further investigated [37,38]. Particularly, anti-suicidal and antidepressant effects of ESK in patients with suicidal ideation might be independent of one another [38]. The ameliorative effects of ketamine for suicide ideation might be less durable than the antidepressant action [39].

## 3. Methods

This systematic review was performed following the Preferred Reporting Items for Systematic Reviews and Meta-Analyses (PRISMA) guidelines [40].

A search was performed in the main psychiatric databases, PubMed (National Li-brary of Medicine), PsychINFO, EMBASE (Ovid), Cochrane Library, to find relevant papers. Moreover, the registries of US NIH (National Institutes of Health, Clinical Tri-als.gov) clinical trials were consulted. Two independent investigators found papers with related abstracts and full texts in English until 31 March 2021 using the following search strategy: “Esketamine (Depressive Disorder OR depression) (nasal OR intranasal)”. The first selection was made using the pertinence of the title and the information in the abstract, the second was made after careful reading of the research methods in the full text.

Two authors subsequently checked and extracted data from included articles: paper author and title, publication year, details of the intervention (dosage, frequency and duration), any adjunctive treatment, type of control used (placebo or other pharmacological intervention), patient characteristics (age, gender, ethnicity or race, diagnosis), primary and secondary outcomes, assessment scales, trial design, trial sample size, duration of follow-up, and publication status. If relevant data were not reported in the selected articles, the corresponding author was contacted to obtain further information. Global rating was performed following criteria by Sterne et al. [33,34]. The strength of clinical data were graded according to the Oxford Centre for Evidence-Based Medicine levels of evidence [41] by 2 authors independently, with discrepancies resolved after joint article review and discussion.

Inclusion criteria consisted of: (1) age of patients ≥18 years; (2) diagnosis of MDD [42]; (3) reduction of MDD symptoms as primary outcome; (4) moving on to the maintenance phase after receiving intranasal esketamine in the initial four-week induction phase; (5) English.

Exclusion criteria were: (1) reviews, meta-analyses, commentaries, letters, case reports, pooled analyses, comments, case studies, study protocols; (2) mixed or inaccurately described diagnoses (e.g., mixed groups with bipolar and unipolar depression, anxiety disorders) unless data were available for the subgroup of MDD patients; (3) studies conducted on animals.

The study protocol was registered in the International Prospective Register of Systematic Reviews (PROSPERO—registration number: CRD42021234017).

## 4. Conclusions

Taken together, the findings of our systematic review on the effectiveness of ESK in the long-term are mixed. Continued ESK treatment following the induction phase may be associated with stable efficacy in relapse prevention among TRD patients. However, the long-term antidepressant and anti-suicidal effects of ESK after discontinuation might be inconsistent. Caution is therefore required in interpreting the available data. More RCTs with larger sample sizes and active comparators are needed to reduce the considerable uncertainty. Moreover, further research is required to elucidate the potential risk of abuse and when and how ESK should be discontinued. Individualization of ESK nasal spray treatment frequency may be a valuable option for optimizing the use of the compound in real world clinical practice [43]. Nevertheless, other molecules with more potent and longer-lasting antidepressant effects than ESK and fewer psychomimetic side effects might be used in clinical practice in place of ESK [10,44,45].

## Figures and Tables

**Table 1:** Summary of the studies on intranasal ESK efficacy in the continuation-treatment phase with ESK after the 4-week induction phase.

Reference	Phase	Sample	Age Mean (SD), y	Design	Country	Dosage	Duration	Risk of Bias	Long-Term Outcome Measures	Results	Cohen’s d
Daly et al., 2018 [26] (1) *	II	57	44.7 (10.04)	Multicenter: -screening; -double-blind treatment (days 1–8 and 8–15): PBO/PBO or PBO/ESK or ESK/ESK -optional open-label treatment (days 15–74) -post-treatment follow-up (days 74–130)	United States and Belgium	Flexible dose Range: 28–84 mg, weekly for 3 weeks, then every 2 weeks	8.5 weeks	High ^1^	MADRS	Response rates after post-treatment follow-up ^a,b^: PBO/PBO: 100% PBO/ESK: 50% ESK/ESK: 61% Remission rates after post treatment follow-up ^a,b^: PBO/PBO: 33% PBO/ESK: 40% ESK/ESK: 28%	Responders: d = −0.185 Remitters: d = −0.245
Daly et al., 2019 [27] SUSTAIN I (1) *	III	297	46.3 (11.13)	Multicenter: -screening and prospective observation phase -open-label induction phase (4 weeks) -fixed-dose optimization phase (12 weeks) -Responders and remitters→ flexible-dose randomized maintenance phase (relapsed during maintenance → long-term safety study) -post-treatment follow-up (2 weeks)	United States, Canada, Europe	Responders: 56 mg or 84 mg once weekly Remitters: 56 mg or 84 mg/2 weeks	Median among responders: 19.4 weeks Median among remitters: 17.7 weeks	Low ^1^	**-Time to** **relapse** **(median)** -MADRS	**Responders ^c^:** **PBO > ESK** **(*p* < 0.001)** PBO: 88.0 days ESK: 635.0 days **Remitters ^c^:** **PBO > ESK** **(*p* = 0.003)** PBO: 273.0 days ESK: NE Relapse rates: -Responders: PBO: 57.6% ESK: 25.8% -Remitters: PBO: 45.3% ESK: 26.7%	Responders: d = −0.288 Remitters: d = −0.171 Responders: d = −0.751 Remitters: d = −0.455
Wajs et al., 2020 [28] SUSTAIN II (2) *	III	150	52.2 (±13.7)	Multicenter, open-label: -screening (4 weeks) -induction phase (4 weeks) -optimization/maintenance phase (48 weeks) -follow-up phase (4 weeks)	United States, United Kingdom, Argentina, Brazil, Europe, Republic of Korea, Malaysia, Mexico, South Africa, Australia, Taiwan, Turkey	Flexible dose range (28-mg, 56-mg, or 84-mg)	48 weeks	Moderate ^2^	-MADRS -PHQ-9 -SDS	MADRS: Responders 76.5% Remitters 58.2% PHQ-9: Responders 74.6% Remitters 47.4% SDS: Responders 63.0% Remitters 39.5%	-

Key: * = Oxford Centre for Evidence-Based Medicine. https://www.cebm.ox.ac.uk/resources/levels-of-evidence/ocebm-levels-of-evidence. Accessed on 26 May 2021. ^1^ = Sterne JAC, Savović J, Page MJ, et al. RoB 2: a revised tool for assessing risk of bias in randomized trials. BMJ. 2019;366:l4898. ^2^ = Sterne JA, Hernán MA, Reeves BC, et al. ROBINS-I: a tool for assessing risk of bias in non-randomized studies of interventions. BMJ. 2016;355:i4919. ^a^ Response = reduction in MADRS total score ≥50%; remission = MADRS total score ≤10; ^b^ All patients were treated with ESK during the optional open-label phase independently from having received placebo during the double-blind initial phase.^c^ Response = reduction in MADRS total score ≥50%; remission = MADRS total score ≤12; relapse = MADRS total score ≥22 for 2 consecutive assessments or hospitalization or both. y: years. ESK = esketamine nasal spray. MADRS = Montgomery–Åsberg Depression Rating Scale. NE = not estimable. PBO = placebo. PHQ-9 = Patient Health Questionnaire—9. RCT = randomized, controlled trial. SD = standard deviation. SDS = Sheehan Disability Scale.

**Table 2:** Summary of the studies on intranasal ESK efficacy in the follow-up phase (TAU) after the 4-week induction phase.

Reference	Phase	Sample	Age Mean (SD), y	Design	Country	Duration	Risk of Bias	Long-Term Outcome Measures	Results	Cohen’s d
Canuso et al., 2018 [30] (1) *	II	44 pts at imminent risk for suicide	35.8 (13.03)	Double-blind RCT: -screening (24–48 h) -double-blind treatment (days 1–25) -post-treatment follow-up (days 26–81)	11 sites at United States	8 weeks	High ^1^	**-MADRS** -BECK Scale for Suicide Ideation -BECK Hopelessness Scale -Remission rates ^a^	**-ESK = PBO (*p* = 0.21)** -ESK = PBO (*p* = 0.84) -ESK = PBO (*p* = 0.35) -PBO = 50% ESK = 59.3%	**d = –0.069** d = –0.257 d = 0.030 d = 0.207
Fu et al., 2020 [31] ASPIRE I (1) *	III	84 from ESK group 80 from PBO group	39.3 (12.91)	Double-blind RCT: -screening (24–48 h) -double-blind treatment (days 1–25) -post-treatment follow-up (days 26–90)	United States, Europe, Asia, South Africa	9 weeks	Some concerns ^1^	-MADRS	Low MADRS scores ESK = PBO (no statistics available)	-
Ionescu et al., 2021 [32] ASPIRE II (1) *	III	81 from ESK group 85 from PBO group	40.8 (13.07)	≈	United States, Canada, Argentina, Brazil, Europe, Turkey	≈	Low ^1^	**-MADRS** -CGI-SS-r	**-low MADRS** and CGI-SS-r **scores** **ESK = PBO** (no statistics available)	-
Daly et al., 2018 [26] (1) *	II	41	44.7 (10.04)	Multicenter: -screening -double-blind treatment (days 1–8 and 8–15): PBO/PBO or PBO/ESK or ESK/ESK -optional open-label treatment (days 15–74) -post-treatment follow-up (days 74–130)	United States and Belgium	8 weeks	High ^1^	MADRS	Response rates ^b^: PBO/PBO: 71% PBO/ESK: 25% ESK/ESK: 68% Remission rates ^b^: PBO/PBO: 57% PBO/ESK: 25% ESK/ESK: 46%	Responders: d = 0.596 Remitters: d = 0.197
NCT02918318 [29] (1) *	II	68	43.4 (10.35)	Double-blind induction phase (4 weeks) -Responders—post-treatment phase (relapsed during post-treatment—open-label induction phase with ESK at flexible dose) -Non-responders—DB Follow-up Phase (4 weeks)	Japan	24 weeks	Low ^1^	Time to relapse (median) in participants with response ^a^	ESK28: 32.0 days ESK56: 26.0 days ESK84: 79.5 days PBO: 91.0 days	--
≈	II	37	≈	≈	Japan	≈	Low ^1^	Time to relapse (median) in participants with remission ^a^	ESK28: 34.0 days ESK56: 52.0 days ESK84: 37.0 days PBO: 30.0 days	--

Key: * = Oxford Centre for Evidence-Based Medicine. https://www.cebm.ox.ac.uk/resources/levels-of-evidence/ocebm-levels-of-evidence. Accessed on 26 May 2021. ^1^ = Sterne JAC, Savović J, Page MJ, et al. RoB 2: a revised tool for assessing risk of bias in randomized trials. BMJ. 2019;366:l4898. ^a^ Response = reduction in MADRS total score ≥50%; remission = MADRS total score ≤12; relapse = MADRS total score ≥22 for 2 consecutive assessments or hospitalization or both. ^b^ Response = reduction in MADRS total score ≥50%; remission = MADRS total score ≤10. ≈ = same as above. ESK = esketamine nasal spray; ESK28 = esketamine at 56 mg/d; ESK56 = esketamine at 56 mg/d; ESK84 = esketamine at 84 mg/d. MADRS = Montgomery–Åsberg Depression Rating Scale. CGI-SS-r = Clinical Global Impression-Severity of Suicidality-revised. PBO = placebo. pts = patients. RCT = randomized, controlled trial. SD = standard deviation. SIBAT = Suicide Ideation and Behavior Assessment Tool. TAU = treatment as usual.
